# Serum Oxidized LDL Levels in Type 2 Diabetic Patients with Retinopathy in Mthatha Region of the Eastern Cape Province of South Africa

**DOI:** 10.1155/2016/2063103

**Published:** 2016-06-28

**Authors:** Farzana Ganjifrockwala, Jim Joseph, Grace George

**Affiliations:** Division of Medical Biochemistry, Department of Human Biology, Faculty of Health Sciences, Walter Sisulu University, Mthatha Campus, Nelson Mandela Drive, Mthatha 5100, South Africa

## Abstract

Oxidized low-density lipoprotein (ox-LDL) is a powerful natural prooxidant derived from native LDL by cell-mediated oxidation. Such oxidation occurs more easily in glycated LDL as observed in diabetes mellitus. We evaluated and compared selected biomarkers of oxidative stress and total antioxidant (TAO) levels in type 2 diabetes mellitus (T2DM) patients with and without retinopathy in the Mthatha region of the Eastern Cape Province, South Africa. The participants totaled to 140 and this number comprised 98 diabetic patients on treatment, stratified by diabetes (54) and diabetes with retinopathy (44). Forty-two nondiabetic healthy controls made up the 140. Fasting plasma glucose (FPG), glycosylated hemoglobin (HbA1c), lipid profile, serum ox-LDL, thiobarbituric acid reactive substances (TBARS), and TAO levels were measured. A statistically significant increase in FPG, HbA1c, TBARS, and ox-LDL and a significant decrease in TAO levels were seen in T2DM patients with retinopathy as compared to controls. A significant negative correlation was observed between TAO and ox-LDL levels in the diabetic group. In multiple linear regression analyses, duration of diabetes, triglyceride, TAO, and LDL cholesterol were found to be significantly associated with ox-LDL. In multiple logistic regression analyses, ox-LDL [OR 1.02 (1.01–1.03), *P* = 0.005] was the only risk factor and was significantly associated with the presence of retinopathy.

## 1. Introduction

Diabetes mellitus is a multifactorial disease often associated with abnormally high blood glucose levels (hyperglycaemia), which results from the body's inability to synthesize insulin or utilize insulin to its full potential [1–3]. The two most common forms are type 1 diabetes mellitus and type 2 diabetes mellitus [4, 5]. Diabetic retinopathy (DR), which is one of the important complications of diabetes mellitus, is a disease of the retina and is a leading cause of acquired blindness in young adults [6]. DR is characterized by gradual and progressive alterations in the vascular system of the retina resulting from chronic hyperglycaemia. The metabolic consequences of chronic hyperglycaemia include sustained hyperglycaemia in retinal vasculature, which leads to the accumulation of advanced glycation end products (AGEs). Inflammation, neuronal dysfunction, changes in redox homeostasis, and oxidative stress are also consequences [2, 7, 8]. Diabetic individuals, both type 1 and type 2, are at risk of developing DR but with type 2 the prevalence is much higher [7]. DR is classified as “background” or “nonproliferative DR” (BDR/NPDR) and “proliferative DR” (PDR) [9–11]. Lipid alteration and oxidizability of lipoproteins are considered to contribute to oxidative stress in diabetes mellitus [12]. The concept of oxidative stress and the role of ox-LDL in atherosclerosis have been known for more than 25 years. ox-LDL was originally defined as “oxidatively modified LDL” that contained protein components “modified” by aldehyde products creating a net negative charge which were essential for its interaction and uptake by macrophages [13]. LDL's chemical composition makes these particles susceptible to oxidation by different lipid oxidants [14]. The precise mechanism of LDL oxidation is not clear, although several mechanisms have been suggested and divided into enzymatic and nonenzymatic processes. The nonenzymatic process involves free transition metal ions such as iron and copper, which play a role in catalyzing lipid peroxidation. The enzymatic process involves a number of different enzyme systems such as lipoxygenases and myeloperoxidase that catalyze the formation of hypochlorous acid, a potent oxidant that modifies apo-B100 at multiple sites [14, 15].

Yu and Lyons [16] hypothesized that ox-LDL may contribute to the pathogenesis of retinopathy through the initial damage of the blood retinal barrier (BRB) along with other metabolic factors commonly seen in diabetes. ox-LDL is not a primary cause of BRB damage, however, and gets involved only once the BRB becomes leaky. Following BRB damage, extravasation of LDL in the extracellular tissue modifies LDL further through oxidation and glycation, making it more toxic towards retinal cells.

It is also well known that the modification of LDL makes it more immunogenic as shown by the formation of LDL-immune complexes (LDL-ICs) and these ox-LDL-ICs exert greater toxicity on retinal capillary pericytes [17].

The present study was undertaken to evaluate oxidative stress and TAO levels in DR patients and whether there is increased lipid peroxidation in DR as compared to diabetes without retinopathy.

## 2. Materials and Methods

Ethics clearance for the study was granted by the Research Ethics Committee of the Faculty of Health Sciences, Walter Sisulu University (reference number: 012/012). Type 2 diabetic patients of Xhosa ethnicity were approached and briefed about the study. These patients fell into the age group of 35–75 years and attended a selected diabetic clinic and ophthalmology referral clinic (Nelson Mandela Academic Hospital (NMAH)) in Mthatha. Those who consented to take part in the study were recruited (patients with HIV infection and other chronic diseases, like tuberculosis, thyroid problems, heart problems, and renal failure if reported, were not included in the study; patients taking multivitamins and statins were also excluded).

A trained clinician at the diabetic clinic carried out the clinical examination of the type 2 diabetic patients. Selected participants were referred to the eye clinic of NMAH where they were screened for retinopathies. At the eye clinic, patients who had dilated pupils were examined through +90 lenses and indirect ophthalmoscope. An ophthalmologist carried out these examinations. Further confirmation and recording of fundus were done with a high-resolution fundus camera. Diabetic retinopathy was classified through early treatment DR study (ETDRS) and was broadly classified as BDR and PDR. Type 2 diabetic patients with and without retinopathy were included in the study after their retinopathy status was confirmed. Control participants were selected by convenience sampling from the general population. Selection criteria were kept in mind throughout the recruitment process.

A total of 140 participants were recruited by the convenience sampling method and were subdivided into three groups. These were healthy controls (42), type 2 diabetics without retinopathy (54), and type 2 diabetics with retinopathy (44) (BDR: 28; PDR: 16).

Anthropometric measurements of height, weight, waist circumference, and hip circumference were taken as a way of assessing the nutritional status and body mass index (BMI). A total of 20 mL of fasting blood was collected in vacutainer tubes. Plasma was separated from the specimen within 3 hours after collection. Serum samples were stored at −70°C if not analyzed on the day they were collected. Fasting blood samples collected from all participants were used for measurements of plasma glucose, HbA1c, and lipid profile on the day they were collected. Thawed serum samples were used within a month for analyzing the TAO, TBARS, and ox-LDL levels.

Roche Cobas 6000 chemical autoanalyzer was used for measuring plasma glucose, glycated hemoglobin, and lipid profile by standard laboratory methods carried out by the National Health Laboratory Services (NHLS) of the NMAH.

The TAO level in serum was measured using a commercial kit from Sigma-Aldrich using the ABTS method. ox-LDL was measured using an ELISA kit from Mercodia (Sweden) and TBARS were analyzed by the trichloroacetic acid (TCA) method using Cayman assay kit. The BioTek KC_4_ Autoreader was used for all analyses referred to above.

Statistical analysis was performed using IBM Statistical Package for the Social Sciences (SPSS Inc., Chicago, IL, USA, version 23). Data were expressed as mean ± SD (standard deviation) and median (IQR). The mean differences between the quantitative variables across groups (control, diabetics, and diabetics with retinopathy) were evaluated by the one-way ANOVA (Analysis of Variance) test. The Tukey HSD (Honest Significant Difference) was used as a* post hoc* test for variables with normal distribution. Nonparametric Kruskal-Wallis test and Dunn's* post hoc* test with Bonferroni's correction were conducted for variables not following normal distribution. Spearman rank correlation was used to analyze relationships between continuous variables. Multivariate linear regression analysis was carried out to check for relationships between various risk factors. Multiple logistic regression analysis was used to calculate the odds ratio for DR with different risk factors.

## 3. Results

The general profile of the participants is presented in Table 1. Age, duration of diabetes, and anthropometric data are presented in Table 2.

The mean age was similar across groups and showed no significant difference (*P* = 0.071). The waist/hip ratio (WHR) was significantly different between groups (*P* < 0.0005) and was higher in diabetic patients with and without retinopathy (median = 0.93 and 0.91, resp.) compared to the healthy controls (median = 0.87). No significant difference in WHR existed among diabetic groups with and without retinopathy (*P* > 0.05). Duration of diabetes was significantly higher in the DR group (median = 10.0 years) compared to the diabetic group without retinopathy (median = 5.0 years, *P* < 0.0005).

Clinical characteristics of participants are presented in Table 3. FPG and HbA1c levels were statistically significantly different between groups (*P* < 0.0005 and *P* < 0.0005), respectively. Diabetic patients with and without retinopathy showed higher FPG and HbA1c levels compared to the healthy control group. The median levels of FPG and HbA1c showed a marginal increase in the DR group as compared to the diabetic group without retinopathy, but the increase was not statistically significant (*P* > 0.05).

Serum triglyceride (TG) (*P* = 0.004) and high-density lipoprotein cholesterol (HDL-C) (*P* = 0.007) showed significant differences among groups. Serum median TG was significantly higher in the DR (1.50 mmol/L) group than in the healthy control group (1.10 mmol/L, *P* = 0.003). The diabetic group without retinopathy did not show any significant differences when compared with the healthy controls and the DR group. Serum median HDL-C was statistically significantly decreased in the diabetic group without retinopathy (1.11 mmol/L, *P* = 0.016) and the DR group (1.18 mmol/L, *P* = 0.017) compared to the control group (1.35 mmol/L). No difference was observed between diabetic groups with and without retinopathy (*P* = 0.931).

A statistically significant decrease in median TAO was observed in the DR group (0.34 mM) compared to the healthy control group (0.60 mM, *P* < 0.0005) and the diabetic group without retinopathy (0.47 mM, *P* = 0.019). The diabetic group without retinopathy also showed a significant decrease in TAO levels as compared to the healthy control group (*P* = 0.048). Significantly higher median TBARS were found in the DR group (5.08 *μ*M) and the diabetic group without retinopathy (4.33 *μ*M) compared to the healthy control group (3.38 *μ*M) (*P* < 0.0005 and *P* = 0.007, resp.). There was a marginal increase in median TBARS levels in the DR group as compared to the diabetic group without retinopathy but this increase was not statistically significant (*P* = 0.556). ox-LDL was significantly increased in the DR group (146.9 ± 56.67 U/L) as compared to the healthy controls (81.76 ± 31.74 U/L, *P* < 0.0005). A significant difference existed in ox-LDL levels in the diabetic group without retinopathy (97.52 ± 40.67 U/L) and the DR group (*P* < 0.0005) but the diabetic group without retinopathy did not show any significant increase in ox-LDL levels when compared to the healthy control group (*P* = 0.199).

**Figure 1:**
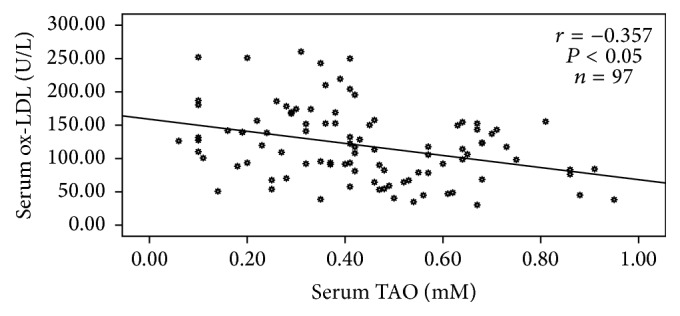
Correlation between TAO and ox-LDL in diabetic group (diabetics with and without retinopathy). Values were considered statistically significant when *P* < 0.05.

**Figure 2:**
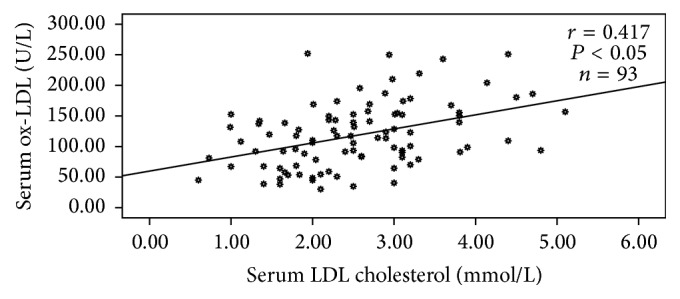
Correlation between LDL cholesterol and ox-LDL in diabetic group (diabetics with and without retinopathy). Values were considered statistically significant when *P* < 0.05.

Correlation analyses between FPG, HbA1c, lipids, TBARS, and ox-LDL in all groups are shown in Table 4. Correlation analysis between ox-LDL and TAO levels showed significant negative correlation in the diabetic group, as seen in Figure 1. This correlation did not show any significance in the healthy control group (*r* = −0.030, *P* = 0.854).

ox-LDL showed significant negative correlation with HDL-C and TBARS and significant positive correlation with low-density lipoprotein cholesterol (LDL-C) and HbA1c in the control group. In the diabetic group, ox-LDL showed a significant positive correlation with TG, total cholesterol (TC), and LDL-C.  Figure 2 shows the correlation between LDL-C and ox-LDL in the diabetic group. No significant correlation was observed with HDL-C, FPG, HbA1c, and TBARS in the diabetic group.

Multiple linear regression analysis was carried out to check for the relationship between ox-LDL and independent variables like age, BMI, FPG, HbA1c, duration of disease, WHR, TG, HDL-C, LDL-C, TBARS, and TAO as shown in Table 5. Only duration of diabetes (*P* = 0.02), TG (*P* = 0.012), TAO (*P* = 0.027), and LDL-C (*P* = 0.001) were found to be significantly associated with ox-LDL levels.

**Figure 3:**
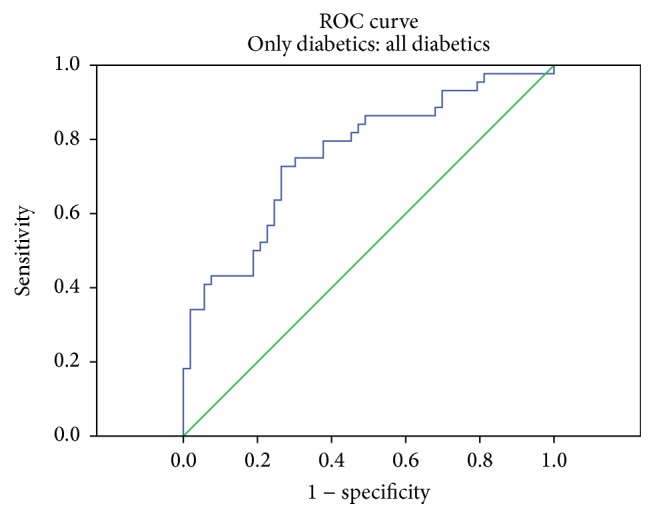
ROC curve for ox-LDL in diabetic groups.

Multiple logistic regression analysis was carried out to check the risk factors for DR, and ox-LDL [OR 1.02 (1.01–1.03), *P* = 0.005] was the only risk factor that was found to be significantly associated with the presence of retinopathy (as shown in Table 6). Age, duration of diabetes, HbA1c, TG, HDL-C, TAO, and TBARS demonstrated no significance. According to the logistic regression analysis, the diagnostic performance of ox-LDL ≥ 115 U/L was the optimal cutoff point. Area under curve (AUC) was 0.760 (95% CI: 0.664–0.857), SE = 0.049, *P* < 0.001. Sensitivity was 75% and specificity was 68% (Figure 3).

## 4. Discussion

A marked increase was found in HbA1c and FPG in the diabetic patients as compared to the control population. Poor diabetic control and excessive glycosylation of hemoglobin, similar to other studies, were indicated [12, 18–21]. The lipid profile showed no difference in TC and LDL-C levels between diabetic patients and control participants, whereas serum TG was higher and HDL-C was lower in diabetic patients, indicating the presence of dyslipidemia. Since insulin mediates the uptake of free fatty acids (FFAs) by skeletal muscle and adipose tissue, an increase in insulin resistance would result in increased FFAs delivered to the liver, which would lead to overproduction of very-low-density lipoprotein (VLDL). Increased VLDL concentration is clinically manifested as hypertriglyceridemia. Decreased lipoprotein lipase activity as a result of insulin resistance would also lead to accumulation of triglyceride-rich lipoproteins in plasma [22]. Hypertriglyceridemia and reduced plasma HDL-C were reported earlier by many authors in diabetes [18, 19, 23].

Chronic hyperglycaemia, elevated FFAs, and dyslipidemia cause oxidative stress by triggering the production of reactive nitrogen species (RNS) and reactive oxygen species (ROS) [24]. These reactive species attack lipids found in plasma as well as the membranes of mitochondria and endoplasmic reticulum. The same species also cause peroxidation [25]. Free radical-induced lipid peroxidation of cellular structures is a chain reaction that provides a continuous supply of free radicals that initiate further peroxidation. Increase in serum TBARS in diabetes is attributed to increased oxidative stress and increased free radical production [14, 19, 20, 26–29]. In our study, serum TBARS was significantly increased in diabetic patients (both with and without retinopathy) as compared to healthy controls as observed by earlier studies [30–36].

Merzouk et al. observed increased LDL oxidizability in diabetic patients irrespective of the type and presence of complications and have attributed it to poor glycaemic control [12]. The poor control was attributed to increased glycation of LDL, which makes it more susceptible to oxidation as reported by an* in vitro* study [37]. TG levels have an effect on LDL size and HDL-C prevents oxidative modification of LDL, so increased TG and decreased HDL-C levels could influence the susceptibility of LDL to oxidation in diabetes [12].

In this study, the serum ox-LDL level was found to be significantly increased in the DR group as compared to the healthy control and the diabetic group without retinopathy. Increased ox-LDL has been reported in type 2 diabetic patients [38, 39] compared to a healthy control group but was not statistically significant. Conversely, a significant increase in ox-LDL in type 2 diabetic patients as compared to a healthy control group was reported by others [14, 40, 41]. In the DR patients, glycaemic control was poor and lipid levels were altered (low HDL-C and high TG), which may be the reason for high ox-LDL levels in the DR group. Serum ox-LDL levels in type 2 diabetic microvascular complications are not much studied, especially in DR.

In the present study, we observed a decrease in TAO levels among diabetic patients and this decrease could be attributed to increased oxidative stress and lipid peroxidation as evidenced by an increase in TBARS and ox-LDL levels. Lipid peroxidation might have resulted in the excess utilization of antioxidants against oxidative stress. Similar observations were reported by Nour Eldin et al. [14], Benrebai et al. [20], and Jamuna Rani and Mythili [27].

In diabetic group(s), ox-LDL showed a significant negative correlation with TAO. Decreased TAO capacity seems to play a role in oxidation of LDL in diabetic patients irrespective of LDL levels. Similar observations were reported by Nour Eldin et al. in type 2 diabetic Saudi men [14].

A significant positive correlation of ox-LDL with TG, TC, and LDL-C in the diabetic group in this study showed a relationship between dyslipidemia and ox-LDL as observed by Nour Eldin et al. [14] and Kopprasch et al. [39].

No significant correlation was observed between HDL-C and ox-LDL in the diabetic group but the control group showed a significant negative correlation. HDL-C is involved in the reverse transport of cholesterol from the periphery to the liver and contains enzymes such as paraoxonase, platelet-activating factor, acetylhydrolase, and lecithin cholesterol acyltransferase. These enzymes prevent the formation of or metabolize the oxidized phospholipids. Evidence exists that HDL can reverse LDL oxidation by removing the oxidized phospholipids that make LDL harmful and lead to atherogenesis [42]. High HDL-C can decrease oxidation of LDL, as observed in our healthy control group, where a significant negative association is observed between the two. In the diabetic group, this correlation was not significant. Tomás et al. [43] reported a decrease in the activity of paraoxonase-1 (PON1) enzyme in type 2 diabetic patients, which confers antioxidant properties to HDL-C. This may possibly be the reason why no correlation was observed between HDL-C and ox-LDL in the diabetic group.

When the various risk factors like age, BMI, WHR, FPG, HbA1c, duration of diabetes, TBARS, TG, HDL-C, LDL-C, and TAO were included in a multiple linear regression analysis, only the duration of diabetes, TG, LDL-C, and TAO status were significantly associated with the ox-LDL levels. This association might indicate that increased TG and LDL in diabetic patients in association with increased oxidative stress and decreased TAO resulted in increased oxidation of LDL-C. Kopprasch et al. also reported LDL-C and TG to be the strongest predictors of circulating ox-LDL levels [39].

Multiple logistic regression showed a significant relationship between ox-LDL levels and the presence of DR. This might probably indicate the involvement of ox-LDL in the pathogenesis and development of DR. In DR, retinal damage results from endothelial cell damage, retina vascular leakage, and lack of perfusion. These processes are known to begin far in advance of any clinical evidence of retinopathy and are associated with increased retinal leukostasis. Leukocyte adhesion to the diabetic vascular endothelium can promote endothelial apoptosis and might be responsible for pericyte loss, one of the earliest changes in the diabetic retina. ox-LDL-containing ICs are likely to play a role in enhancing retinal leukostasis. Other possible mechanisms by which ox-LDL-containing ICs may contribute to DR are stimulation of growth factors, like vascular endothelial growth factor, and production of matrix proteins, factors which lead to thickening of the retinal vascular basement membrane, a well-known characteristic of DR [44].

## 5. Conclusion

In summary, ox-LDL is significantly increased and TAO was significantly decreased in diabetic patients with retinopathy as compared to diabetics without retinopathy and healthy controls. A negative correlation with TAO and a positive correlation with LDL-C and TG show that dyslipidemia and low total antioxidants may play a role in oxidation of native LDL to ox-LDL. ox-LDL ≥ 115 U/L might be considered an important risk factor for DR in this population.

## Figures and Tables

**Table 1:** Profile of research participants in different groups.

Variables	Groups			
	Control (nondiabetic) (*n* = 42)	Diabetic without retinopathy (*n* = 54)	DR (*n* = 44)	Total% (*N* = 140)
Gender				
Female	*n* = 27 (19.3%)	*n* = 32 (22.9%)	*n* = 28 (20.0%)	62.1%
Male	*n* = 15 (10.7%)	*n* = 22 (15.7%)	*n* = 16 (11.4%)	37.9%
Hypertension	N/A	*n* = 44 (44.9%)	*n* = 29 (29.6%)	74.5%
Physical activity	*n* = 26 (18.6%)	*n* = 32 (22.9%)	*n* = 24 (17.1%)	58.6%
Alcohol consumption	*n* = 7 (5.0%)	*n* = 2 (1.4%)	*n* = 2 (1.4%)	7.9%
Smoking	*n* = 9 (6.4%)	*n* = 0 (0%)	*n* = 3 (2.1%)	8.6%
Family history	N/A	*n* = 26 (26.5%)	*n* = 30 (30.6%)	57.1%
Medication				
Oral drugs	N/A	*n* = 47 (48.0%)	*n* = 22 (22.4%)	70.4%
Insulin	N/A	*n* = 7 (7.1%)	*n* = 22 (22.4%)	29.6%

DR: diabetic retinopathy.

**Table 2:** Mean age, duration of diabetes, and anthropometric data in different groups.

Variables	Groups			
	Control (nondiabetic) (*n* = 42)	Diabetic without retinopathy (*n* = 54)	DR (*n* = 44)	*P* value
Age (years)	54.67 ± 6.76	56.50 ± 7.56	58.52 ± 8.66	0.071
Duration of diabetes (years)	N/A	5.0 (8.25–2.0)	10.0 (15.0–5.0)	<0.0005^*∗∗∗*^
BMI (Kg/m^2^)	29.7 (35.1–25.2)	31.3 (36.6–26.0)	31.7 (35.7–26.2)	0.618
Weight (Kg)	77.0 (87.5–65.8)	80.0 (95.2–69.5)	80.0 (92.7–69.2)	0.331
Height (cm)	160.0 (163.5–155.5)	162.0 (168.5–155)	160.0 (165–155)	0.249
Waist circumference (cm)	97.5 (106–80.5)	99.0 (109–91)	100.0 (108.5–91)	0.135
Hip circumference (cm)	107.5 (122–93.8)	106.5 (118.2–99)	105.5 (116–99.5)	0.859
Waist/hip ratio	0.87 (0.89–0.85)	0.91 (0.94–0.87)	0.93 (0.95–0.89)	<0.0005^*∗∗∗*^

Data is shown as mean ± SD (standard deviation) and median and IQR (interquartile range). The mean and median difference is significant at ^*∗∗∗*^ *P* < 0.0005 level. DR: diabetic retinopathy.

**Table 3:** Clinical characteristics in different groups.

Variables	Groups			
	Control (nondiabetic) (*n* = 42)	Diabetic without retinopathy (*n* = 54)	DR (*n* = 44)	*P* value
FPG (mmol/L)	4.9 (5.2–4.6)	8.1 (10.5–6.3)	9.6 (13.1–6.5)	<0.0005^*∗∗∗*^
HbA1c (%)	6.0 (6.1–5.8)	8.1 (10.2–6.8)	9.0 (11.1–8.0)	<0.0005^*∗∗∗*^
Total cholesterol (mmol/L)	4.80 (5.37–3.87)	4.50 (5.05–3.67)	4.55 (5.37–3.83)	0.438
LDL cholesterol (mmol/L)	2.75 ± 0.88	2.51 ± 0.96	2.65 ± 1.01	0.490
HDL cholesterol (mmol/L)	1.35 (1.52–1.10)	1.11 (1.40–1.0)	1.18 (1.36–0.91)	0.007^*∗*^
Triglyceride (mmol/L)	1.10 (1.52–0.90)	1.30 (2.0–1.0)	1.50 (2.54–1.11)	0.004^*∗∗*^
Total antioxidant (mM)	0.60 (0.80–0.44)	0.47 (0.61–0.37)	0.34 (0.45–0.23)	<0.0005^*∗∗∗*^
TBARS (*μ*M)	3.38 (4.62–2.32)	4.33 (6.0–3.3)	5.08 (6.12–4.0)	<0.0005^*∗∗∗*^
Serum oxidized LDL (U/L)	81.76 ± 31.74	97.52 ± 40.67	146.9 ± 56.67	<0.0005^*∗∗∗*^

Data is shown as median and IQR (interquartile range) and mean ± SD. The median and mean difference is significant at ^*∗*^ *P* < 0.05, ^*∗∗*^ *P* < 0.005, and ^*∗∗∗*^ *P* < 0.0005 level. DR: diabetic retinopathy.

**Table 4:** Spearman's correlation coefficients between lipid parameters, FPG, HbA1c, TBARS, and ox-LDL in all groups.

Groups	TC *r*-value	LDL-C *r*-value	HDL-C *r*-value	Triglyceride *r*-value	FPG *r*-value	HbA1c *r*-value	TBARS *r*-value
ox-LDL in control group	0.279	0.470^*∗*^	−0.593^*∗*^	0.179	0.198	0.309^*∗*^	−0.469^*∗*^
ox-LDL in diabetic group	0.424^*∗*^	0.417^*∗*^	−0.002	0.293^*∗*^	0.053	0.040	0.134

Data is presented as Spearman correlation coefficient (*r*), ^*∗*^ *P* < 0.05. TC: total cholesterol; HDL-C: high-density lipoprotein cholesterol; LDL-C: low-density lipoprotein cholesterol; FPG: fasting plasma glucose; HbA1c: glycosylated hemoglobin; ox-LDL: oxidized LDL.

**Table 5:** Multiple linear regression analyses showing relationship between ox-LDL and various parameters.

Model	*B*	SE	Beta	*P* value sig.
1 (constant)	−91.539	104.241		0.383
Age	.906	.630	.140	0.155
BMI	1.123	.747	.152	0.137
DD	2.170	.914	.244	0.020^*∗*^
FPG	.880	1.458	.070	0.548
HbA1c	−1.921	2.501	−.092	0.445
TAO	−58.877	26.105	−.225	0.027^*∗*^
TBARS	.015	2.548	.001	0.995
WHR	76.139	77.907	.092	0.332
TG	16.962	6.593	.273	0.012^*∗*^
HDL-C	−1.480	20.097	−.008	0.941
LDL-C	17.692	5.310	.327	0.001^*∗*^

*B* (SE): unstandardized regression coefficient with standard error; Beta: standardized regression coefficient.

BMI: body mass index; DD: duration of diabetes; FPG: fasting plasma glucose; HbA1c: glycosylated hemoglobin; HDL-C: high-density lipoprotein cholesterol; LDL-C: low-density lipoprotein cholesterol; TAO: total antioxidant; ox-LDL: oxidized LDL; TBARS: thiobarbituric acid reactive substances; WHR: waist/hip ratio; TG: triglycerides. ^*∗*^ *P* < 0.05.

**Table 6:** Independent markers associated with diabetic retinopathy among diabetic patients and using multiple logistic regression analysis.

		*B*	SE	Wald	Sig.	Exp (*B*)	95% CI for Exp (*B*)	
							Lower	Upper
Step 1^a^	Age	.019	.034	.319	.572	1.020	.953	1.091
	HbA1c	.181	.116	2.460	.117	1.199	.956	1.504
	DD	.092	.055	2.783	.095	1.096	.984	1.222
	TG	.076	.322	.056	.813	1.079	.574	2.030
	HDL-C	–.728	1.095	.442	.506	.483	.056	4.131
	TAO	–1.840	1.423	1.672	.196	.159	.010	2.584
	Ox-LDL	.018	.006	7.861	.005^*∗*^	1.018	1.005	1.030
	TBARS	–.010	.131	.006	.936	.990	.766	1.279
	Constant	–4.055	3.006	1.820	.177	.017		

^a^Variable(s) entered on step 1: age, HbA1c, DD, TG, HDL, TAO, ox-LDL, and TBARS.

DD: duration of diabetes; HbA1c: glycosylated hemoglobin; TG: triglycerides; HDL-C: high-density lipoprotein cholesterol; TAO: total antioxidant; ox-LDL: oxidized LDL; TBARS: thiobarbituric acid reactive substances. ^*∗*^ *P* < 0.05.
